# The prognostic power of inflammatory indices and clinical factors in metastatic castration-resistant prostate cancer patients treated with radium-223 (BIO-Ra study)

**DOI:** 10.1007/s00259-021-05550-6

**Published:** 2021-09-06

**Authors:** Matteo Bauckneht, Sara Elena Rebuzzi, Alessio Signori, Viviana Frantellizzi, Veronica Murianni, Elisa Lodi Rizzini, Manlio Mascia, Valentina Lavelli, Maria Isabella Donegani, Marta Ponzano, Angela Gaudiano, Maria Lina Stazza, Maria Licari, Letizia Cavallini, Viola Laghi, Luca Cindolo, Martina Maggi, Alessandro Sciarra, Paolo Mammucci, Gianmario Sambuceti, Renato Patrizio Costa, Angela Spanu, Giuseppe Rubini, Fabio Monari, Giuseppe De Vincentis, Giuseppe Fornarini

**Affiliations:** 1 Nuclear Medicine, IRCCS Ospedale Policlinico San Martino, Genova, Italy; 2 Department of Health Sciences (DISSAL), University of Genova, Genova, Italy; 3 Medical Oncology Unit 1, IRCCS Ospedale Policlinico San Martino, Genova, Italy; 4 Department of Internal Medicine and Medical Specialties, University of Genova, Genova, Italy; 5 Department of Radiological Sciences, Oncology and Anatomical Pathology, Sapienza University of Rome, Rome, Italy; 6 Radiation Oncology, IRCCS Azienda Ospedaliero-Universitaria Di Bologna, Bologna, Italy; 7 Unit of Nuclear Medicine, Spirito Santo Hospital, Pescara, Italy; 8 Nuclear Medicine Section, Interdisciplinary Department of Medicine, University of Bari “Aldo Moro”, Bari, Italy; 9 Unit of Nuclear Medicine, Department of Medical, Surgical and Experimental Sciences, University of Sassari, Sassari, Italy; 10 Unit of Nuclear Medicine, Biomedical Department of Internal and Specialist Medicine, University of Palermo, Palermo, Italy; 11 Department of Experimental Diagnostic and Specialty Medicine, Alma Mater Studiorum Bologna University, Bologna, Italy; 12 Department of Urology, Villa Stuart Private Hospital, Rome, Italy; 13 Department of Urology, Sapienza University of Rome, Policlinico Umberto I, Rome, Italy

**Keywords:** Metastatic castration-resistant prostate cancer, [^223^Ra]RaCl_2_, Inflammatory indices, Neutrophil-to-lymphocyte ratio, Clinical factors

## Abstract

**Purpose:**

To combine peripheral blood indices and clinical factors in a prognostic score for metastatic castration-resistant prostate cancer (mCRPC) patients treated with radium-223 dichloride ([^223^Ra]RaCl_2_).

**Patients and methods:**

Baseline neutrophil-to-lymphocyte ratio (NLR), derived NLR (donor), lymphocyte-to-monocyte ratio (LMR), platelet-to-lymphocyte ratio (PLR), systemic inflammation index (SII), Eastern Cooperative Oncology Group performance status (ECOG PS), Gleason score (GS) group, number of bone metastases, prostate-specific antigen (PSA), alkaline phosphatase (ALP), line of therapy, previous chemotherapy, and the presence of lymphadenopathies were collected from seven Italian centers between 2013 and 2020. Lab and clinical data were assessed in correlation with the overall survival (OS). Inflammatory indices were then included separately in the multivariable analyses with the prognostic clinical factors. The model with the highest discriminative ability (c-index) was chosen to develop the BIO-Ra score.

**Results:**

Five hundred and nineteen mCRPC patients (median OS: 19.9 months) were enrolled. Higher NLR, dNLR, PLR, and SII and lower LMR predicted worse OS (all with a *p* < 0.001). The multivariable model including NLR, ECOG PS, number of bone metastases, ALP, and PSA (c-index: 0.724) was chosen to develop the BIO-Ra score. Using the Schneeweiss scoring system, the BIO-Ra score identified three prognostic groups (36%, 27.3%, and 36.6% patients, respectively) with distinct median OS (31, 26.6, and 9.6 months, respectively; hazard ratio: 1.62, *p* = 0.008 for group 2 vs. 1 and 5.77, *p* < 0.001 for group 3 vs. 1).

**Conclusions:**

The BIO-Ra score represents an easy and widely applicable tool for the prognostic stratification of mCRPC patients treated with [^223^Ra]RaCl_2_ with no additional costs.

**Supplementary Information:**

The online version contains supplementary material available at 10.1007/s00259-021-05550-6.

## Introduction

Bone metastases represent the leading cause of poor quality of life and increased mortality in patients with metastatic castration-resistant prostate cancer (mCRPC) [1, 2]. Radium-223 dichloride ([^223^Ra]RaCl_2_) is an alpha-emitting radioisotope that selectively binds to increased osteoblastic activity areas, inducing double-stranded DNA breaks impairing cellular repair mechanisms [3].

The phase III Alpharadin in Symptomatic Prostate Cancer Patients (ALSYMPCA) study demonstrated a significantly prolonged overall survival (OS) and time to first symptomatic skeletal event in mCRPC patients with bone metastasis and no visceral metastatic involvement receiving [^223^Ra]RaCl_2_ compared to placebo [4]. According to these results, [^223^Ra]RaCl_2_ was approved by the Food and Drug Administration in 2013 [5].

However, in later years, the clinical experience has revealed a lower survival benefit than ALSYMPCA results. Several studies reported a median OS (mOS) ranging from 6 to 10 months, which was lower than that reported by the ALSYMPCA trial (14.9 months) [6–8]. Previous studies suggested that this is partially due to a suboptimal selection of patients with unfavorable prognostic characteristics [9]. Moreover, in 2018 the European Medicine Agency (EMA) restricted the use of [^223^Ra]RaCl_2_ to patients with more than six osteoblastic lesions progressing to at least two systemic therapies for mCRPC or ineligible for any systemic mCRPC treatment [10]. The consequent delay of [^223^Ra]RaCl_2_ administration in the later phases of the disease might negatively affect OS, making the patient’s selection process even further challenging [11]. In this scenario, identifying prognostic factors potentially able to select patients most likely to benefit from [^223^Ra]RaCl_2_ since baseline has become a crucial issue.

Cancer-associated inflammation plays a key role in therapeutic response and survival in cancer patients across different tumor types [12]. Many research groups have investigated the predictive and prognostic role of peripheral blood inflammatory parameters, such as the neutrophil-to-lymphocyte ratio (NLR), in different solid tumors [13–17].

A preliminary monocentric study investigated the prognostic role of baseline peripheral blood inflammatory indices focusing on mCRPC patients treated with [^223^Ra]RaCl_2_ showing that increased NLR at baseline identifies patients at higher risk for unfavorable outcomes [18]. With the BIO-Ra study, we extended this analysis to a multicentric setting, aiming to develop a composite prognostic score potentially able to improve the patients’ selection process.

## Materials and methods

The study was performed according to the Declaration of Helsinki, Good Clinical Practice, and local ethical regulations. The local ethical committee of the leading center approved the study (Regional Ethical Committee of Liguria—registration number 535/2020). The study was then approved by the local ethical committee of each adhering center. All patients enrolled in the study signed written informed consent before each [^223^Ra]RaCl_2_ administration, which included the use of anonymized data for retrospective research purposes.

### Study population

A multicenter analysis was conducted on seven Italian centers retrospectively collecting clinical data and laboratory parameters of mCRPC patients receiving [^223^Ra]RaCl_2_ in a real-world setting. CRPC was defined as a serum testosterone level of < 50 ng/dL following surgical or pharmaceutical castration. According to the standard selection criteria for [^223^Ra]RaCl_2_ treatment, patients must have a diagnosis of mCRPC with symptomatic bone metastases and neither visceral metastases nor lymph nodes > 3 cm in short-axis diameter [19]. To be included in the study, patients must have received at least one cycle of [^223^Ra]RaCl_2_.

### Treatment

[^223^Ra]RaCl_2_ (50–55 KBq/kg) was intravenously administrated every 4 weeks and was continued until disease progression, death, or patient choice up to six cycles. Eventual toxicities were managed according to the current guidelines [19]. Treatment with either chemotherapy, abiraterone, or enzalutamide was discontinued before the first [^223^Ra]RaCl_2_ administration. Patients continued receiving androgen deprivation therapy [19].

### Systemic inflammation indices

Complete blood count as assessed soon before the first [^223^Ra]RaCl_2_ administration collecting white blood cells (WBC), platelets (PLT) and the absolute neutrophil (ANC), lymphocyte (ALC), and monocyte (AMC) count. From the complete blood count, we calculated the following ratios: NLR, derived NLR (dNLR), lymphocyte-to-monocyte ratio (LMR), platelets-to-lymphocyte ratio (PLR), and systemic inflammation index (SII). dNLR was calculated as ANC/(WBC-ANC) and SII as NLRxPLT.

### Cut-off determination of the systemic inflammation indices

As many thresholds have been explored, but none validated in mCRPC patients (especially in those treated with [^223^Ra]RaCl_2_), the cut-off values of inflammatory indices were determined using time-dependent ROC curves with the Liu approach, maximizing the concordance probability function [20–23]. The ROC curve was calculated at the time point corresponding to the mOS. An internal validation using 500-times bootstrap resampling was performed. For each index, the optimal cut-off and the c-index were reported.

### Study endpoint

The primary endpoint was overall survival (OS), which was defined as the time from first [^223^Ra]RaCl_2_ administration until death from any cause, censored at last follow-up for patients who were alive.

### Statistical analyses

The descriptive analyses were conducted using absolute frequency and percentage for categorical variables and by median and range for quantitative variables. The Kaplan–Meier (KM) method was used to estimate the survival curve of OS [24]. Differences were considered statistically significant when the *p*-value (*p*) was < 0.05. Univariable and multivariable analyses were performed using Cox proportional hazard regression model, estimating hazard ratios (HRs), and their 95% confidence interval (CI). In the univariable analyses, clinical and laboratory parameters were assessed in correlation with OS. Only factors with a *p* < 0.05 at the univariable analysis were evaluated in the multivariable analyses for OS. Due to a strict correlation among the inflammatory indices, those predictive of OS were included separately in the multivariable analyses together with the clinical factors. Only factors with a *p* < 0.05 in the multivariable analysis were kept in the multivariable model. For each multivariable model, the discriminatory ability as defined by Harrell’s c-index was calculated: a higher c-index represented a better capability of the multivariable model to separate patients with and without the event. A 500-times bootstrap resampling with replacement guaranteed the stability of the c-index. Missing values for indices were imputed (see Supplementary Materials for details). The multivariable model with the highest c-index was chosen for being the basis of the prognostic score. All statistical analyses were performed using the software Stata v.16 (StataCorp 2019) and R v.4.0.2 [25, 26].

### Prognostic score

The selection procedure for the prognostic score and the parameter estimation from the Cox model was internally validated using the bootstrap approach (see Supplementary Materials for details).

To consider the possible overfitting during building and estimation of the prognostic score, a bias-corrected estimate of the discriminatory ability (c-index) was calculated with 500 bootstrap samples using the Design package in R.

The prognostic score was calculated using the regression coefficient based (Schneeweiss) scoring system. The weight assigned to each factor in the score was defined based on the Cox regression model’s regression coefficient [27].

Finally, the prognostic score was stratified in risk strata according to the likelihood-ratio test and after checking the score’s survival estimates.

### Dynamic alkaline phosphatase (ALP) change

The association of each determinant included in the BIO-Ra score and the score itself with the dynamic alkaline phosphatase (ALP) change was investigated using a linear regression model with the dynamic ALP change as the dependent variable. Dynamic ALP change was defined as the percentage change at week 12, after the third cycle of [223Ra]RaCl_2_, the halfway point of treatment, as previously described [28]. Results were reported as linear regression coefficients with 95% confidence intervals.

## Results

### Patients’ characteristics

Five hundred and nineteen mCRPC patients were included in the analysis. Patients’ and treatment characteristics are summarized in Table 1. The median age was 74 years (range: 50–90 years), and patients with ≥ 75 years were 48% of the entire cohort. At the time of [^223^Ra]RaCl_2_ initiation, most patients had an Eastern Cooperative Oncology Group performance status (ECOG PS) of 0–1 (77%), no lymph nodal metastases (61%), and a number of bone metastases between 6 and 20 (57%). Among all patients, 48% received [^223^Ra]RaCl_2_ as first- and second-line therapy, while 52% as further lines, while most of them had previously received chemotherapy (62%).

**Table 1:** Patients’ characteristics

Patients *n* = 519	
Characteristics	*N* (%)
Age, years	
Median (range)	74 (50–90)
< 75	268 (52)
≥ 75	251 (48)
Gleason score	
Median (range)	8 (5–10)
≤ 7	173 (33)
≥ 8	263 (51)
Missing	83 (16)
ECOG PS	
Median (range)	1 (0–3)
0–1	399 (77)
2–3	120 (23)
[^223^Ra]RaCl_2_ treatment line	
Median (range)	3 (1–9)
First and second line	249 (48)
≥ Third line	270 (52)
Prior chemotherapy	
Yes	324 (62)
No	195 (38)
Lymphadenopathies	
Yes	161 (31)
No	317 (61)
Missing	41 (8)
N bone metastases	
< 6	60 (12)
6–20	295 (57)
> 20	155 (30)
Missing	9 (2)
Bisphosphonates/denosumab use	
Yes	233 (45)
No	283 (55)
Missing	3 (1)
[^223^Ra]RaCl_2_ treatment before/after EMA restriction	
After	98 (19)
Before	421 (81)
Baseline ALP, U/L	
Median (range)	142 (0–2474)
< 220	340 (66)
≥ 220	167 (32)
Missing	12 (2)
Baseline median LDH, U/L (range)	294 (129–2146)
Baseline median PSA, ng/ml (range)	54.0 (0.03–6089)
Baseline median Hb, g/dL (range)	12.2 (7.8–16.4)

*ECOG* Eastern Cooperative Oncology Group, *EMA* European Medicines Agency, *ALP* alkaline phosphatase, *LDH* lactate dehydrogenase, *PSA* prostate-specific antigen, *Hb* hemoglobin

### Results in the overall population

At the time of data cut-off (February 2021), with a median follow-up of 10.7 months, 251 patients (48%) were dead and the mOS was 19.9 months (95% CI 17.7–23.8), and the completion rates of the first three and six cycles were 88% and 63%, respectively.

### Identification of the cut-off values

Bone scan lesions were categorized in < 6, 6–20, and > 20, as previously described [29]. According to the current guidelines [19], patients with advanced diffuse metastatic infiltration of the bone (“superscan”) should be treated with [223Ra]RaCl_2_ only after a careful benefit-risk assessment. Due to this subgroup’s low statistical power, we included these patients in the third category. The cut-off values identified for the inflammatory indices were 3.1 for NLR, 2.0 for dNLR, 2.8 for LMR, 146 for PLR, and 769 for SII, while for prostate-specific antigen (PSA) was 44 ng/mL. For each inflammatory index and PSA, the median value, the optimal cut-off, and the c-index are shown in Supplementary Table 1.

### Univariable analyses for OS

**Fig. 1:**
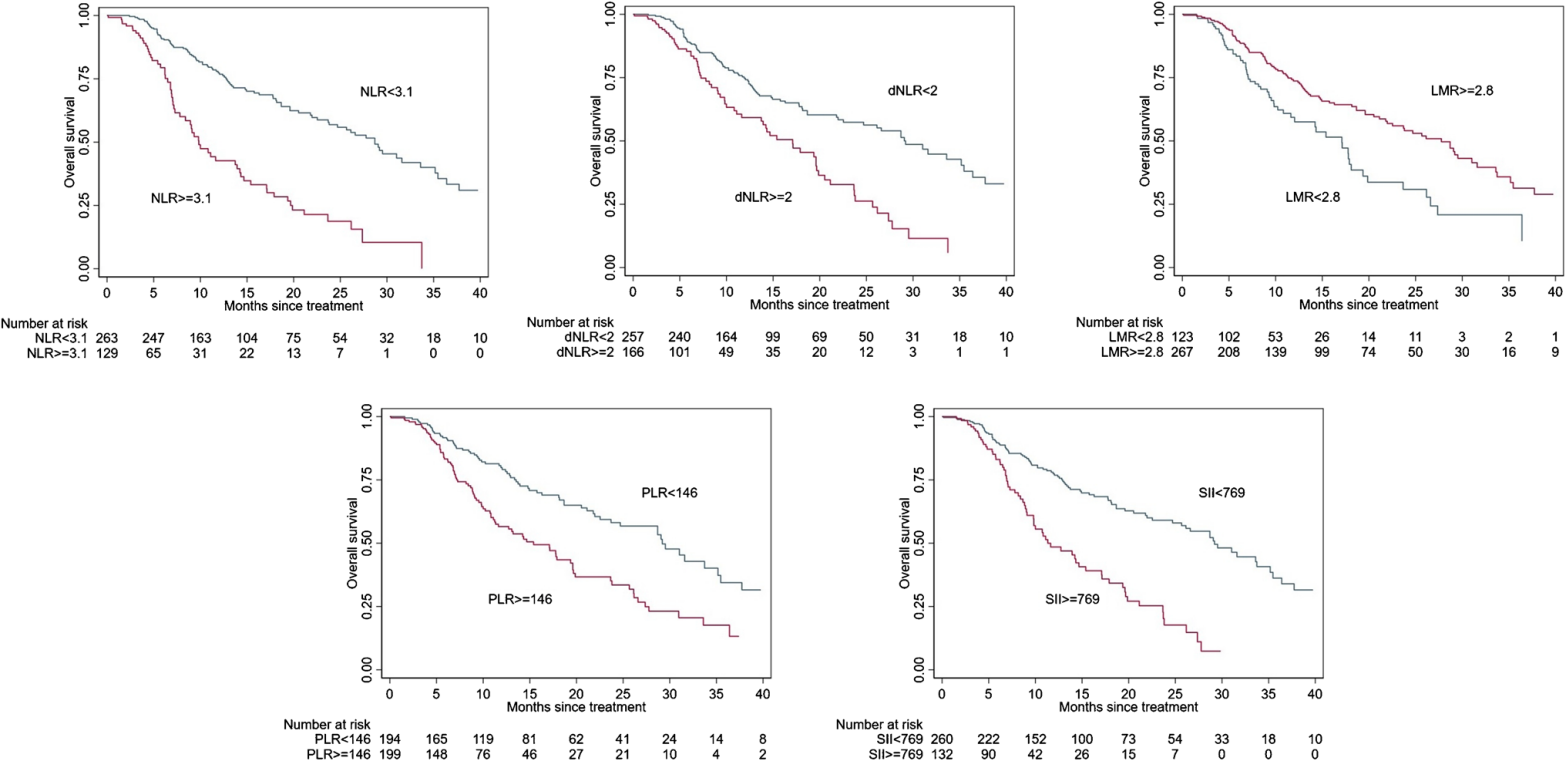
Kaplan–Meier survival curves according to the systemic inflammatory indices. OS prediction according to neutrophil-to-lymphocyte ratio (NLR), derived-NLR (d-NLR), lymphocyte-to-monocyte ratio (LMR), platelets-to-lymphocyte ratio (PLR), and systemic inflammation index (SII)

**Fig. 2:**
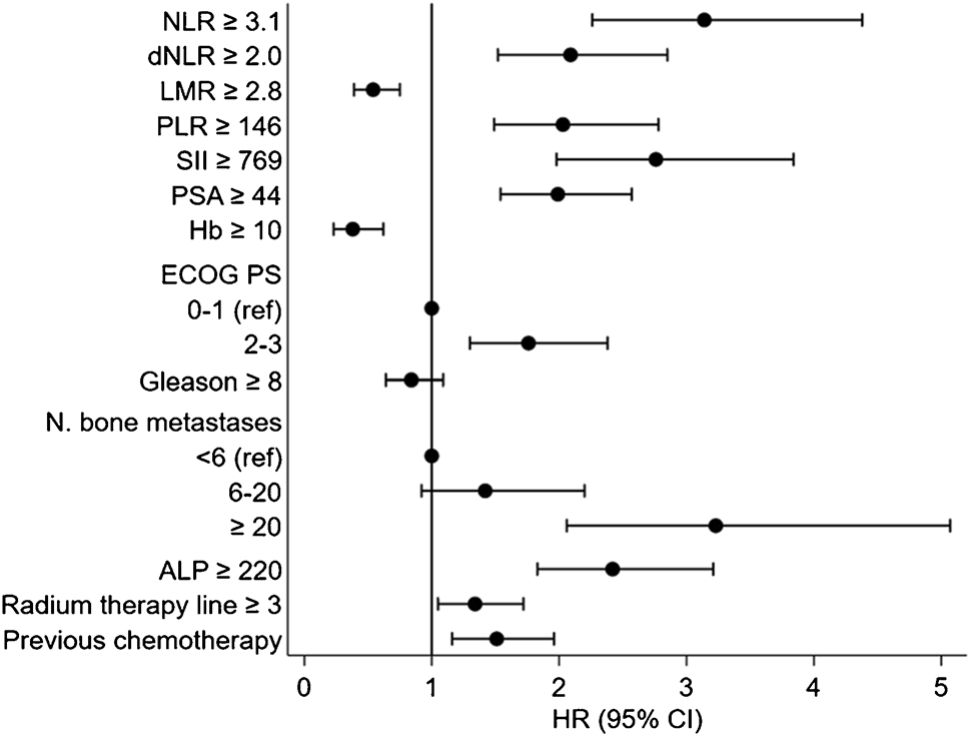
Forest plot of the HRs obtained at the univariable analyses

Results from univariable analyses are reported in Table 2. All biomarkers and clinical factors, except for Gleason score (GS) group (defined as < 8 or ≥ 8) and the presence of lymphadenopathies, significantly correlated with OS at the univariable analyses (Table 2). Specifically, among inflammatory indices, higher NLR, dNLR, PLR, and SII were associated with worse OS, while higher LMR predicted longer OS (all with a *p* < 0.001) (Fig. 1). The univariable analyses have been graphically summarized in one forest plot (Fig. 2).

**Table 2:** Univariable Cox regression analyses

	*N*	*HR (95% CI)*	*p value*
NLR	392		** < 0.001**
< 3.1	263	1.00 (ref)	
≥ 3.1	129	3.14 (2.26–4.38)	
dNLR	423		** < 0.001**
< 2.0	257	1.00 (ref)	
≥ 2.0	166	2.09 (1.52–2.85)	
LMR	390		** < 0.001**
< 2.8	123	1.00 (ref)	
≥ 2.8	267	0.54 (0.39–0.75)	
PLR	393		** < 0.001**
< 145.9	194	1.00 (ref)	
≥ 145.9	199	2.03 (1.49–2.78)	
SII	392		** < 0.001**
< 768.8	260	1.00 (ref)	
≥ 768.8	132	2.76 (1.98–3.84)	
ECOG PS	519		** < 0.001**
0–1	399	1.00 (ref)	
2–3	120	1.76 (1.30–2.38)	
Gleason score group	439		0.189
< 8	173	1.00 (ref)	
≥ 8	266	0.84 (0.64–1.09)	
**N** bone metastases	510		
< 6	60	1.00 (ref)	
6–20	295	1.42 (0.92–2.20)	0.116
≥ 20	155	3.23 (2.06–5.07)	** < 0.001**
**ALP, U/L**	507		** < 0.001**
< 220	340	1.00 (ref)	
≥ 220	167	2.42 (1.83–3.21)	
**ALP, U/L**			** < 0.001**
≤ 140	249	1.00 (ref)	
> 140	258	1.95 (1.51–2.53)	
[^223^Ra]RaCl_2_ therapy line	519		**0.021**
1–2	251	1.00 (ref)	
≥ 3	268	1.34 (1.05–1.72)	
Previous chemotherapy	519		**0.002**
No	195	1.00 (ref)	
Yes	324	1.51 (1.16–1.96)	
PSA, ng/mL	515		** < 0.001**
< 44	231	1.00 (ref)	
≥ 44	284	1.99 (1.54–2.57)	
Hb, g/dL	518		** < 0.001**
< 10	31	1.00 (ref)	
≥ 10	487	0.38 (0.23–0.62)	

*NRL* neutrophil–lymphocyte ratio, *dNLR* derived neutrophil–lymphocyte ratio, *LMR* lymphocyte-to-monocyte ratio, *PLR* platelet-to-monocyte ratio, *SII* systemic immune-inflammation index, *ECOG* Eastern Cooperative Oncology Group, *ALP* alkaline phosphatase, *PSA* prostate-specific antigen, *Hb* hemoglobin

Bold indicates significant *p* values

### Multivariable analyses for OS

Among 519 patients of the entire cohort, 494 patients (95%) were evaluable for multivariable analyses after multiple imputations of missing inflammation indices. In the multivariable analyses, all inflammatory indices, ECOG PS, the number of bone metastases, ALP, and PSA were confirmed as independent predictors of survival.

The multivariable model with NLR (< 3.1 vs. ≥ 3.1), ECOG PS (0–1 vs. 2–3), number of bone metastases (< 6, 6–20, > 20), ALP (< 220 vs. ≥ 220), and PSA (< 44 vs. ≥ 44) showed the highest discriminative ability (c-index: 0.724) (Table 3). For this reason, this multivariable model was chosen for the development of the prognostic score. Results were consistent with the analyses on complete cases (Supplementary Table 2).

**Table 3:** Multivariable Cox regression analyses on OS

	*N*	*HR (95% CI)*	*p-value*		*N*	*HR (95% CI)*	*p-value*		*N*	*HR (95% CI)*	*p-value*		*N*	*HR (95% CI)*	*p-value*		*N*	*HR (95% CI)*	*p-value*	
***c-index***				**0.7235**				**0.7061**				**0.6951**				**0.7001**				**0.7151**
**NLR**	494		** < 0.001**																	
< 3.1		1.00 (ref)																		
≥ 3.1		2.70 (2.06–3.56)																		
**dNLR**					494		** < 0.001**													
< 2.0						1,00 (ref)														
≥ 2.0						2.22 (1.70–2.91)														
**LMR**									494		** < 0.001**									
< 2.8										1.00 (ref)										
≥ 2.8										0.56 (0.42–0.75)										
**PLR**													494		** < 0.001**					
< 145.9														1.00 (ref)						
≥ 145.9														1.80 (1.38–2.35)						
**SII**																	494		** < 0.001**	
< 768.8																		1.00 (ref)		
≥ 768.8																		2.40 (1.82–3.16)		
**ECOG PS**			**0.039**				**0.017**				0.190				0.065				0.086	
0–1		1.00 (ref)				1.00 (ref)				1.00 (ref)				1.00 (ref)				1.00 (ref)		
2–3		1.40 (1.02–1.93)				1.48 (1.07–2.03)				1.24 (0.90–1.73)				1.35 (0.98–1.86)				1.32 (0.96–1.83)		
**N** **bone metastases**																				
< 6		1.00 (ref)	–-			1.00 (ref)				1.00 (ref)	–-			1.00 (ref)	–-			1.00 (ref)	–-	
6–20		1.18 (0.75–1.85)	0.464			1.22 (0.78–1.91)	0.391			1.15 (0.73–1.80)	0.546			1.11 (0.71–1.74)	0.651			1.19 (0.76–1.87)	0.442	
≥ 20		2.14 (1.32–3.46)	**0.002**			2.22 (1.36–3.60)	**0.001**			2.31 (1.42–3.75)	**0.001**			2.03 (1.26–3.28)	**0.004**			2.07 (1.28–3.36)	**0.003**	
**ALP**			** < 0.001**				** < 0.001**				** < 0.001**				** < 0.001**				** < 0.001**	
< 220		1.00 (ref)				1.00 (ref)				1.00 (ref)				1.00 (ref)				1.00 (ref)		
≥ 220		2.03 (1.50–2.73)				2.00 (1.48–2.69)				1.85 (1.37–2.50)				2.03 (1.51–2.73)				2.03 (1.50–2.74)		
**PSA**																				
< 44		1.00 (ref)	**0.040**			1.00 (ref)	**0.047**			1.00 (ref)	**0.012**			1.00 (ref)	**0.021**			1.00 (ref)	**0.014**	
≥ 44		1.36 (1.01–1.81)				1.35 (1.00–1.80)				1.46 (1.09–1.95)				1.40 (1.05–1.86)				1.44 (1.08–1.93)		

Bold indicates significant p values

*NRL* neutrophil–lymphocyte ratio, *dNLR* derived neutrophil–lymphocyte ratio, *LMR* lymphocyte-to-monocyte ratio, *PLR* platelet-to-monocyte ratio, *SII* systemic immune-inflammation index, *ECOG* Eastern Cooperative Oncology Group, *ALP* alkaline phosphatase, *PSA* prostate-specific antigen

### Prognostic score

After 500 bootstrap replications, in 276 replications (55.2%), all five prognostic factors were included, while in 204 (40.8%), four prognostic factors were maintained. The prognostic factors alternatively excluded were the PSA or the ECOG PS, while NLR, number of bone metastases, and ALP were kept in more than 95% of models. The regression parameters and HRs calculated from the 500 bootstrap samples were remarkably similar to those obtained from the original Cox model, suggesting an excellent internal validation (Table 4). The bias-corrected c-index for optimism from possible overfitting was 0.717 by the bootstrap procedure.

**Table 4:** Bootstrap internal validation of multivariable Cox regression coefficients and risk scoring definition

	Original set (N = 494)	Bootstrap (500 replication)		Risk-scoring (Schneeweiss)
	β-coefficient ± SE	β-coefficient ± SE	HR (95% CI)	
Parameter				
NLR				
< 3.1	0	0	1.00 (ref)	0
≥ 3.1	0.99 ± 0.14	1.02 ± 0.15	2.77 (2.07–3.72)	3
ECOG-PS				
0–1	0	0	1.00 (ref)	0
2–3	0.34 ± 0.16	0.35 ± 0.18	1.42 (1.00–2.02)	1
N bone metastases				
< 6	0	0	1.00 (ref)	0
6–20	0.17 ± 0.23	0.18 ± 0.22	1.20 (0.78–1.84)	1
≥ 20	0.76 ± 0.25	0.79 ± 0.24	2.20 (1.38–3.53)	3
ALP, U/L				
< 220	0	0	1.00 (ref)	0
≥ 220	0.71 ± 0.15	0.71 ± 0.16	2.03 (1.49–2.78)	2
PSA, ng/mL				
< 44	0	0	1.00 (ref)	0
≥ 44	0.30 ± 0.15	0.34 ± 0.15	1.40 (1.05–1.89)	1
Harrell’s c-index	0.7235	0.7173*		
Optimism		0.006		

^*^Optimism-corrected value

*NRL* neutrophil–lymphocyte ratio, *ECOG* Eastern Cooperative Oncology Group, *ALP* alkaline phosphatase, *PSA* prostate-specific antigen

**Fig. 3:**
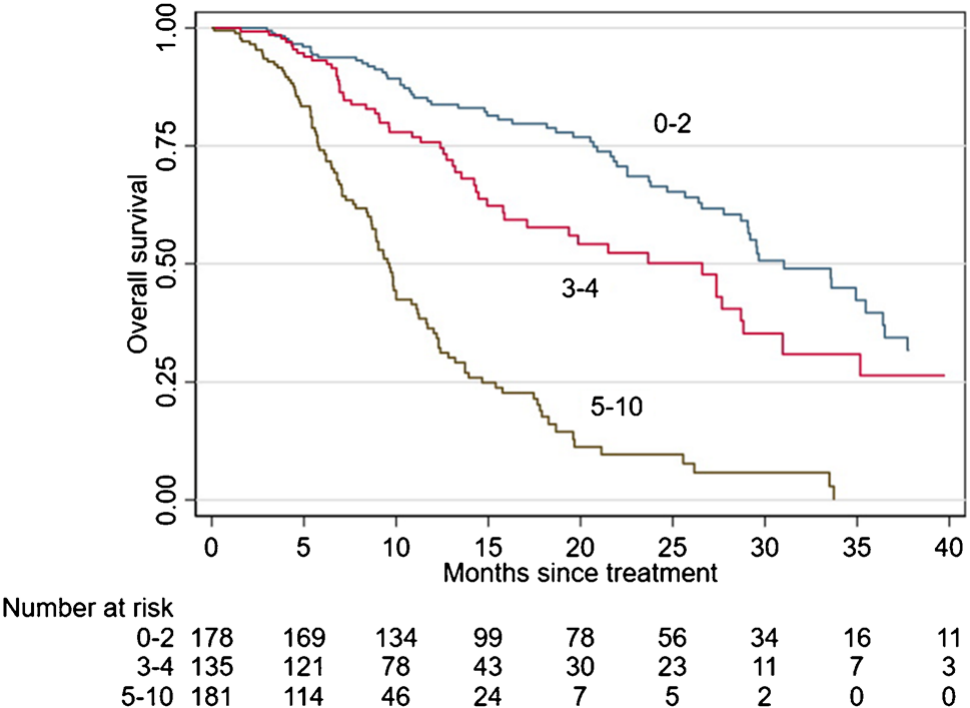
Kaplan–Meier survival curves according to the BIO-Ra score. OS prediction according to the BIO-Ra score, categorizing patients into three prognostic groups (score 0–2, 3–4, and 5–10, showing mOS of 31.0, 26.6, and 9.6 months, respectively)

The point assignation according to the bootstrapped Cox model coefficients and the Schneeweiss scoring system was reported and ranged from a minimum of 0 to a maximum of 10 points. The final prognostic score, called “BIO-Ra score,” had a c-index of 0.723. After application of the survival ROC curve and for a better clinical interpretation, the ten prognostic classes were combined in three prognostic groups characterized by very distinctive OS: the prognostic group 1 (score 0–2), the prognostic group 2 (score 3–4), and the prognostic group 3 (score 5–10) (Fig. 3). According to the BIO-Ra score (Fig. 3), the prognostic group 1 (178 patients, 36%) had a significantly longer mOS (31.0 months) compared to the prognostic group 2 (135 patients, 27.3%) (26.6 months, HR 1.62, *p* = 0.008) and the prognostic group 3 (181 patients, 36.6%) (mOS: 9.6 months, HR = 5.77, *p* < 0.001).

### Dynamic ALP change

ALP values at baseline and at week 12 were both available in 383 patients. The mean ALP change was of − 7.6% (SD: 159; median: − 23.1; IQR: − 41.7, − 3.8). Neither of the determinants of the BIO-Ra score showed significant associations with the dynamic ALP change: NLR (*p* = 0.77), ECOG PS (*p* = 0.56), bone metastases (6–20 vs. < 6: *p* = 0.39, > 20 vs. < 6, *p* = 0.86), ALP (*p* = 0.092), and PSA (*p* = 0.17). Similarly, the three-level BIO-Ra score (3–4 and 5–10 vs. 0–2) was not associated with dynamic ALP change (coefficients of − 8.7, 95% CI =  − 47.8, 30.4; *p* = 0.66, and − 16.2, 95% CI =  − 54.4, 22.1; *p* = 0.41, respectively).

## Discussion

[^223^Ra]RaCl_2_ is the first bone seeking radiopharmaceutical showing to both provide bone palliation and improve OS [4]. In the last years, it has become a valuable therapeutic option for mCRPC patients with symptomatic bone metastases. However, lower survival outcomes have been reported in real life [6], compared to the OS reported in the pivotal phase III ALSYMPCA trial [4].

This weaker survival benefit may be related to the lack of standardization of the optimal timing, sequence, and combinations of [^223^Ra]RaCl_2_ with other therapeutic agents for mCRPC [9]. Moreover, there are no approved prognostic or predictive factors to identify mCRPC patients who would most benefit from [^223^Ra]RaCl_2_ [30]. However, a growing amount of scientific data suggests that the efficacy of [^223^Ra]RaCl_2_ is strictly dependent on pre-treatment patients’ prognostic stratification [9].

Several studies investigated many potential baseline prognostic factors, whose application might optimize the patient’s selection process. In the present multicenter retrospective study, we observed that baseline ECOG PS, PSA and ALP levels, the number of bone metastases, and NLR provide prognostic insights in a large cohort of mCRPC patients receiving [^223^Ra]RaCl_2_. These findings align with the literature since all these parameters represent established prognostic factors in this clinical setting. ECOG PS represents one of the most validated and reproducible tools for assessing the overall clinical status and one of the most important prognostic factors in treating advanced tumors, including mCRPC [31]. In fact, mCRPC patients treated with [^223^Ra]RaCl_2_ show significantly longer survival outcomes in the presence of ECOG PS 0 than ECOG PS 1 or 2 [31]. Moreover, while the ALSYMPCA trial exclusively included patients with painful bone metastases [4], more recent data showed that OS tended to be longer in patients with little or no bone pain than those with moderate or severe pain [31].

On the other hand, many studies already showed the prognostic value of circulating PSA and ALP at baseline, as biochemical indicators of the tumor extent [28, 31–38]. This is coherent with the unfavorable prognosis observed in patients with massive bone metastases (> 20) at the bone scan or high tumor burden assessed with more sophisticated quantification approaches [32, 39–43].

Altogether, these data support the emerging notion that utilizing [^223^Ra]RaCl_2_ early on in the course of mCRPC represents a reasonable and effective strategy [44]. Indeed, the administration of [^223^Ra]RaCl_2_ before the ECOG PS declines to 2 or worse might increase the chances to complete the therapeutic scheme, which is crucial for deriving the maximal benefit from the therapy [4]. On the other hand, higher PSA or ALP values and higher bone metastases are related to a higher tumor burden, which carries a reduced response rate [42].

Given the consolidated role of cancer-related inflammation in tumorigenesis, prognosis, and response to oncological therapies [12], we extended our analysis to peripheral blood inflammatory indices. As a whole reflecting an inflamed state, these blood parameters have been investigated as potential prognostic and predictive factors in different settings [15]. In the last years, a few studies showed the predictive value of NLR in mCRPC patients treated with [^223^Ra]RaCl_2_, related to the prediction of PFS and OS [6, 35]. A recent monocentric study analyzed many inflammatory indices as prognostic factors in this clinical setting, confirming that NLR is the most accurate survival predictor among inflammatory indices [18].

**Fig. 4:**
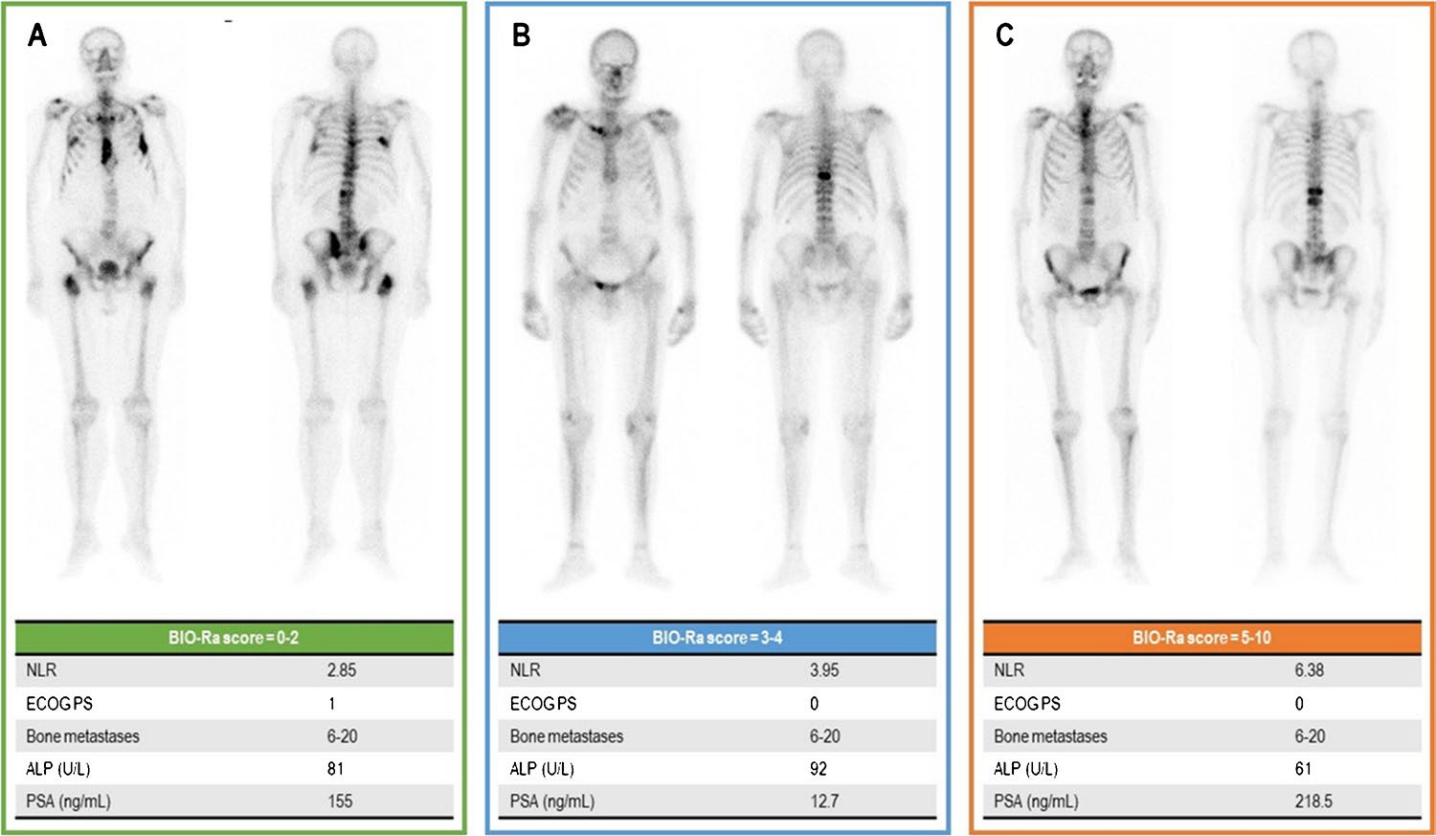
Emblematic cases belonging to the three BIO-Ra classes. Panels **A**–**C** represent the bone scans of three emblematic cases belonging to the three BIO-Ra classes (BIO-Ra score 0–2, 3–4, and 5–10, respectively). The clinical and biochemical determinants of the BIO-Ra score are reported in each panel as well

However, most of the studies mentioned above remained inconclusive since none of these clinical or biochemical parameters has been validated as a unique and reliable selection tool. Consequently, in real-world clinical practice, we still encounter mCRPC patients with rapid disease progression after the [^223^Ra]RaCl_2_ administration. With the BIO-Ra study, we developed a composite score combining performance status, tumor burden, and systemic inflammation, which can identify a subgroup of mCRPC patients who most likely benefit from [^223^Ra]RaCl_2_ therapy. Its determinants are widely available in the clinical routine making the final prognostic score a broadly applicable tool for clinical practice with no additional costs. Emblematic cases belonging to the three prognostic categories are represented in Fig. 4.

Two previous studies have proposed integrating three prognostic biomarkers to obtain a 3-variable predicting score based on baseline ECOG PS, PSA, and Hb serum levels in mCRPC patients treated with [^223^Ra]RaCl_2_ [8, 45]. These studies were conducted on 92 and 430 patients, respectively. Compared with the larger study on 430 patients, the BIO-Ra study includes the ALP value and the number of bone metastases improving its reliability for stratifying the extent of the tumor burden compared to the sole PSA [46]. Moreover, the inclusion of NLR makes it able to describe at the same time the degree of systemic inflammation, which plays an essential role in the prognosis and response to therapies regardless of the extent of the tumor burden [18]. On the methodological ground, a robust approach based on bootstrap was used to define the optimal cut-off for each indices, to stabilize the c-index and the regression coefficients, and in general to consistently validate the obtained score.

Of note, even if prognostic, the baseline BIO-Ra score was not correlated with the biochemical response to therapy (measured as the dynamic change of ALP). Even if validated hematologic biomarkers for monitoring treatment response in mCRPC receiving [^223^Ra]RaCl_2_ are currently lacking, a few studies reported that the ALP dynamic change might predict treatment benefit [28, 47, 48]. However, a post hoc analysis of the ALSYMPCA trial showed that while a reduction in ALP from baseline to week 12 significantly reduced the risk of death, proportional treatment effect values based on Cox regression models did not show the surrogacy for OS [28]. On these bases, it is currently not recommended to discontinue [^223^Ra]RaCl_2_ therapy on the sole basis of dynamic ALP changes. It is reasonable that introducing a combinatory set of variables might better stratify treatment response concerning the only ALP change. Future studies might incorporate a combination of clinical and laboratory biomarkers when evaluating [^223^Ra]RaCl_2_ treatment response. According to this, we are currently planning to analyze the prognostic power of the BIO-Ra score dynamic change in [^223^Ra]RaCl_2_-treated patients.

The present study has some limitations. First, due to the multicentric nature of the study, even if made according to the current guidelines [19], the location of [^223^Ra]RaCl_2_ in the therapeutic sequence with the other life-prolonging agents for mCRPC was chosen by each participating center, thus potentially introducing biases. Second, these results are someway preliminary, as they would need validation on an independent patient population. Furthermore, a prospective randomized trial is needed to confirm the capability of the BIO-Ra score in identifying patients who would most benefit from [^223^Ra]RaCl_2_ treatment, thus optimizing the patient’s selection process. In particular, the preserved survival benefit of [^223^Ra]RaCl_2_ treatment in the presence of higher BIO-Ra risk classes needs to be clarified. As a final consideration, the cohort of 519 mCRPC patients was exclusively treated with [^223^Ra]RaCl_2_ plus androgen deprivation therapy. Thus, we cannot assume the generalizability of the BIO-Ra score’s prognostic role to [^223^Ra]RaCl_2_-based treatment combinations that are currently under investigation.

## Conclusion

In the BIO-Ra study, we investigated the prognostic role of clinical factors and inflammatory indices and their combination in a prognostic score in mCRPC patients receiving [^223^Ra]RaCl_2_ therapy. The BIO-Ra score allows an accurate prognostic stratification of mCRPC patients treated with [^223^Ra]RaCl_2_, providing an easy and widely applicable tool for clinical practice at no additional costs. Future plans include the external validation of the prognostic value, the assessment of its predictivity, and its application in patients receiving [^223^Ra]RaCl_2_-based treatment combinations.

## Supplementary Information

Below is the link to the electronic supplementary material.

Supplementary file1 (DOCX 27.3 KB)

## Data Availability

The data that support the findings of this study are available from the corresponding author upon reasonable request.
