# Supplementary material for: The prognostic power of inflammatory indices and clinical factors in metastatic castration-resistant prostate cancer patients treated with radium-223 (BIO-Ra study)
Source: Eur J Nucl Med Mol Imaging. 2021 Sep 6;49(3):1063–74. doi: 10.1007/s00259-021-05550-6 (PMC8803683; doi:10.1007/s00259-021-05550-6)
Supplement: Supplementary file 1 — Supplementary file1 (DOCX 27.3 KB) [file 259_2021_5550_MOESM1_ESM.docx]

**Supplementary methods**

*Missing values*

The multiple imputation approach by chained equations (MICE) was used to impute missing values of systemic inflammation indices. Particularly, ten iterations of the algorithm were performed. The variables used in the model for imputation were age, adjuvant RT, hemoglobin, and PSA with the complete blood counts (WBC, PLT, ANC, ALC, AMC) as dependent variables.

*Prognostic score*

The first step to create the prognostic score was to internally validate the selection procedure for the multivariable Cox regression model for OS. From the original sample, 500 bootstrap samples with replacement were randomly generated and the number of times that each laboratory parameter and clinical characteristic had been introduced in the multivariable model was calculated. All variables included in more than 50% of the models were confirmed in the prognostic score. In the second step, the internal validation of parameter estimates from the Cox model was performed. Five hundred bootstrap samples were randomly generated from the original sample and for each one the Cox regression model with the selected variables was run and the regression coefficient, the standard error (SE) and the HR with the 95% CI were calculated.

**Supplementary Table 1.** Optimal cutpoints and c-index for PSA and inflammation indexes

|  | | | | |
| --- | --- | --- | --- | --- |
|  | ***N*** | ***Median (range)*** | ***Optimal Cutpoint*** | ***C-index*** |
| **NLR** | 392 | 2.7 (0.6-115.6) | 3.1 | 0.63 |
| **dNLR** | 423 | 1.9 (0.1-7.5) | 2.0 | 0.60 |
| **LMR** | 390 | 3.2 (0.6-22.3) | 2.8 | 0.65 |
| **PLR** | 393 | 146.2 (20.2-5320) | 146 | 0.61 |
| **SII** | 392 | 588.0 (69.8- 30749.6) | 769 | 0.60 |
| **PSA** | 515 | 54.0 (0-6089) | 44 | 0.65 |

*NRL* neutrophil-lymphocyte ratio*, dNLR* derived neutrophil-lymphocyte ratio, *LMR* lymphocyte-to-monocyte ratio**,** *PLR* platelet-to-monocyte ratio, *SII* systemic immune-inflammation index, *PSA* prostate-specific antigen.

| **Supplementary Table 2.** Multivariable Cox regression analyses on OS using complete cases patients | | | | | | | | | | | | | | | | | | | | |
| --- | --- | --- | --- | --- | --- | --- | --- | --- | --- | --- | --- | --- | --- | --- | --- | --- | --- | --- | --- | --- |
|  | ***N*** | ***HR (95% CI)*** | ***p-value*** | ***c-index*** | ***N*** | ***HR (95% CI)*** | ***p-value*** |  | ***N*** | ***HR (95% CI)*** | ***p-value*** |  | ***N*** | ***HR (95% CI)*** | ***p-value*** |  | ***N*** | ***HR (95% CI)*** | ***p-value*** |  |
| **c-index** |  |  |  | 0.7235 |  |  |  | 0.6959 |  |  |  | 0.6993 |  |  |  | 0.7105 |  |  |  | 0.7138 |
| **NLR** | 381 |  | **<0.001** |  |  |  |  |  |  |  |  |  |  |  |  |  |  |  |  |  |
| <3.1 |  | 1.00 (ref) |  |  |  |  |  |  |  |  |  |  |  |  |  |  |  |  |  |  |
| ≥3.1 |  | 2.98 (2.11-4.20) |  |  |  |  |  |  |  |  |  |  |  |  |  |  |  |  |  |  |
| **dNLR** |  |  |  |  | 411 |  | **<0.001** |  |  |  |  |  |  |  |  |  |  |  |  |  |
| <2.0 |  |  |  |  |  | 1,00 (ref) |  |  |  |  |  |  |  |  |  |  |  |  |  |  |
| ≥2.0 |  |  |  |  |  | 2.04 (1.47-2.82) |  |  |  |  |  |  |  |  |  |  |  |  |  |  |
| **LMR** |  |  |  |  |  |  |  |  | 379 |  | **<0.001** |  |  |  |  |  |  |  |  |  |
| <2.8 |  |  |  |  |  |  |  |  |  | 1.00 (ref) |  |  |  |  |  |  |  |  |  |  |
| ≥2.8 |  |  |  |  |  |  |  |  |  | 0.54 (0.38-0.76) |  |  |  |  |  |  |  |  |  |  |
| **PLR** |  |  |  |  |  |  |  |  |  |  |  |  | 382 |  | **<0.001** |  |  |  |  |  |
| <145.9 |  |  |  |  |  |  |  |  |  |  |  |  |  | 1.00 (ref) |  |  |  |  |  |  |
| ≥145.9 |  |  |  |  |  |  |  |  |  |  |  |  |  | 2.04 (1.47-2.82) |  |  |  |  |  |  |
| **SII** |  |  |  |  |  |  |  |  |  |  |  |  |  |  |  |  | 381 |  | **<0.001** |  |
| <768.8 |  |  |  |  |  |  |  |  |  |  |  |  |  |  |  |  |  | 1.00 (ref) |  |  |
| ≥768.8 |  |  |  |  |  |  |  |  |  |  |  |  |  |  |  |  |  | 2.56 (1.81-3.62) |  |  |
| **ECOG PS** |  |  | **0.009** |  |  |  | **0.014** |  |  |  | **0.046** |  |  |  | **0.010** |  |  |  | **0.037** |  |
| 0-1 |  | 1.00(ref) |  |  |  | 1.00(ref) |  |  |  | 1.00(ref) |  |  |  | 1.00(ref) |  |  |  | 1.00(ref) |  |  |
| 2-3 |  | 1.64(1.13-2.37) |  |  |  | 1.56(1.09-2.23) |  |  |  | 1.47(1.01-2.16) |  |  |  | 1.63(1.13-2.36) |  |  |  | 1.49(1.02-2.17) |  |  |
| **N bone metastases** |  |  |  |  |  |  |  |  |  |  |  |  |  |  |  |  |  |  |  |  |
| <6 |  | 1.00(ref) | --- |  |  | 1.00(ref) |  |  |  | 1.00(ref) | --- |  |  | 1.00(ref) | --- |  |  | 1.00(ref) | --- |  |
| 6-20 |  | 1.02 (0.59-1.73) | 0.956 |  |  | 1.03(0.61-1.75) | 0.913 |  |  | 0.89 (0.52-1.53) | 0.682 |  |  | 0.79(0.47-1.35) | 0.398 |  |  | 0.99(0.58-1.68) | 0.958 |  |
| >=20 |  | 2.04(1.12-3.71) | **0.019** |  |  | 2.19(1.22-3.91) | **0.008** |  |  | 2.03(1.11-3.69) | **0.021** |  |  | 1.59(0.87-2.90) | 0.133 |  |  | 1.84(1.02-3.35) | **0.044** |  |
| **ALP** |  |  | **0.002** |  |  |  | **0.001** |  |  |  | **0.017** |  |  |  | **0.002** |  |  |  | **0.003** |  |
| <220 |  | 1.00(ref) |  |  |  | 1.00(ref) |  |  |  | 1.00(ref) | --- |  |  | 1.00(ref) |  |  |  | 1.00(ref) |  |  |
| >=220 |  | 1.80(1.24-2.62) |  |  |  | 1.85(1.29-2.64) |  |  |  | 1.56(1.08-2.26) |  |  |  | 1.81(1.25-2.63) |  |  |  | 1.77(1.22-2.58) |  |  |
| **PSA** |  |  | 0.391 |  |  |  | 0.113 |  |  |  | 0.073 |  |  |  | 0.086 |  |  |  | 0.101 |  |
| < 44 |  | 1.00(ref) |  |  |  | 1.00(ref) |  |  |  | 1.00(ref) |  |  |  | 1.00(ref) |  |  |  | 1.00(ref) |  |  |
| >= 44 |  | 1.18(0.81-1.73) |  |  |  | 1.34(0.93-1.93) |  |  |  | 1.41(0.97-2.06) |  |  |  | 1.38(0.96-1.98) |  |  |  | 1.37(0.94-1.99) |  |  |
